# A unique influenza A (H5N1) virus causing a focal poultry outbreak in 2007 in Manipur, India

**DOI:** 10.1186/1743-422X-6-26

**Published:** 2009-02-24

**Authors:** Akhilesh C Mishra, Sarah S Cherian, Alok K Chakrabarti, Shailesh D Pawar, Santosh M Jadhav, Biswajoy Pal, Satish Raut, Santosh Koratkar, Sadhana S Kode

**Affiliations:** 1 Microbial Containment Complex, National Institute of Virology, Sus Road, Pashan, Pune 411021, Maharashtra, India; 2 National Institute of Virology, 20-A, Dr Ambedkar Road, Pune 411001, India

## Abstract

**Background:**

A focal H5N1 outbreak in poultry was reported from Manipur, a north-eastern state, of India, in 2007. The aim of this study was to genetically characterize the Manipur isolate to understand the relationship with other H5N1 isolates and to trace the possible source of introduction of the virus into the country.

**Results:**

Characterization of the complete genome revealed that the virus belonged to clade 2.2. It was distinctly different from viruses of the three EMA sublineages of clade 2.2 but related to isolates from wild migratory waterfowl from Russia, China and Mongolia. The HA gene, had the cleavage site GERRRRKR, earlier reported in whooper swan isolates from Mongolia in 2005. A stop codon at position 29 in the PB1-F2 protein could have implications on the replication efficiency. The acquisition of polymorphisms as seen in recent isolates of 2005–07 from distinct geographical regions suggests the possibility of transportation of H5N1 viruses through migratory birds.

**Conclusion:**

Considering that all eight genes of the earlier Indian isolates belonged to the EMA3 sublineage and similar strains have not been reported from neighbouring countries of the subcontinent, it appears that the virus may have been introduced independently.

## Background

Highly pathogenic avian influenza (HPAI) A – H5N1 viruses have now appeared in about 60 countries causing devastating outbreaks in poultry with continued capacity to impact humans [1]. The virus was initially isolated from geese in Guangdong, China in 1996 [2]. The Hong Kong reassortant viruses that infected human in 1997 [3] were eliminated due to massive culling of poultry, but the ancestors remained and generated various new genotypes [4]. The virus that re-emerged in South Korea in late 2003 [5] spread to south-east Asian countries [6]. Another major emergence was noticed after an outbreak in migratory birds in Qinghai lake, western China, in 2005, [7] causing outbreaks in many countries in Europe, Middle-East, Africa and Asia [8,9]. The virus is continuously evolving and diversifying into different clades. All the viruses that caused outbreaks in China, Europe, Middle-Eastern and African regions grouped into genotype Z, clade 2.2 [10,11]. The isolates from India and Bangladesh perhaps form the south-eastern geographical boundary for this clade. The clade includes 3 sublineages namely EMA 1 to 3 [12] and some unassigned viruses.

The first outbreak of the H5N1 virus in India was reported from Maharashtra in January 2006 [13]. Seven episodes in poultry were recorded up to April 2006 in the western states of Maharashtra, Gujarat and central Madhya Pradesh. Complete genome sequencing of the H5N1 isolates of 2006 revealed that all eight genes belonged to the sublineage EMA3 of the clade 2.2. The close similarity of the virus to geographical regions of the East Africa/West-Asian and Central Asian migratory bird flyways, suggested that the virus in India might have been introduced through migratory birds [14]. In July 2007, a small outbreak of H5N1 in poultry was reported from Manipur, a north-eastern state, of India. The outbreak was controlled and no spread was noted in the neighbouring areas.

The aim of this study was to genetically characterize the Manipur isolate of 2007 to understand the relationship with other H5N1 isolates and to trace the possible source of introduction of the virus into the country.

## Materials and methods

**Figure 1:**
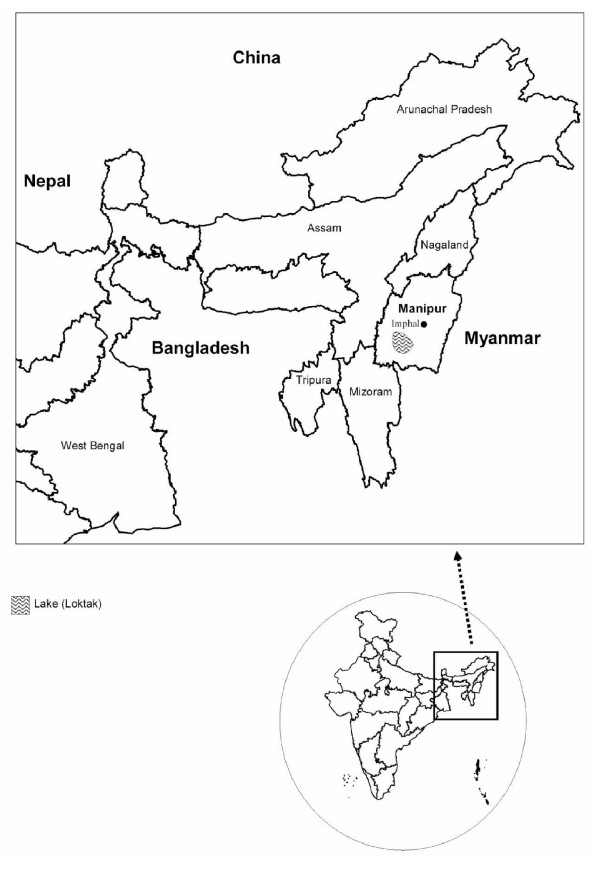
**Map showing the location of the H5N1 outbreak in the state of Manipur, India**.

The state of Manipur (latitude 23°83'N – 25°68'N and longitude 93°03'E – 94°78'E) is known for some animal sanctuaries that are home to many exotic flora and fauna (Figure 1). A large number of migratory birds visit Loktak, an ecologically rich freshwater lake dotted with floating islands, about 45 km away from the capital city, Imphal. The city lies in a valley of ~700 sq. miles surrounded by mountains at an elevation of 790 metres above sea level. The outbreak was reported in Chingmeirong, East Imphal. The capital is well connected to Myanmar in the east and to Bangladesh in the west both of which reported Avian Influenza outbreaks in 2006–07.

### Virus Isolation

Six clinical samples from different organs (trachea, lung, spleen, liver, heart and kidney) of a sick bird were received from Manipur. Specimens were processed for virus isolation in specific-pathogen-free (SPF) embryonated chicken eggs and Madin Darby Canine Kidney (MDCK) cell lines as described earlier [14]. Inoculated eggs were observed for 24–48 hours before harvesting the allantoic fluid. All experiments using infectious virus were conducted in a biosafety level 3 (enhanced) laboratory.

### Identification

Hemagglutination (HA) and Hemagglutination inhibition (HAI) tests were performed as described by Kendal et al. [15]. Horse Red blood cells (1.0% suspension) were used for the HA and HAI test. The reference antisera used were influenza A(H5N1)-NIV/Navapur, H5N1-WHO, H5N2, H9N2, H7N3, and Newcastle disease virus (obtained from the OIE reference laboratory, Venice, Italy).

RNA was extracted using QIAamp Viral RNA Minikit (QIAGEN, Germany) following manufacturers instruction. One-Step reverse transcription-PCR (RT-PCR) was performed using the QIAGEN one-step RT-PCR kit and WHO recommended diagnostic primer sets specific for influenza A HA (H5) and NA (N1) genes [16]. RNA isolated from the specimens was tested by Real Time RT-PCR. Applied Biosystems' TaqMan Influenza A/H5 Detection Kit Version 1.0 was used on Applied Biosystems' 7300 Real-Time PCR platform. Negative controls were processed along with the specimens to rule out cross contamination.

### Whole genome sequencing

RNA isolated from the specimens was reverse transcribed as mentioned earlier [14]. cDNA was used to amplify all the eight gene segments using segment specific primers [17]. The PCR products were purified from an agarose gel using gel extraction kit (QIAGEN,) and amplicons were directly sequenced using an automated 3130 XL Genetic analyzer (Applied Biosystems).

### Phylogenetic analysis

For phylogenetic analysis, representative sequences of the H5N1 viruses belonging to the Z genotype were selected from the GenBank based on sequence identity (100% identical sequences were excluded) and geographical representation. In this process of selection, the sequences whose whole genome was available were preferred. Phylogenetic analysis was performed using the Bayesian approach for tree construction as implemented in Mr Bayes 3.2 [18]. The GTR (General Time Reversible) + I (Invariable sites) model with gamma-distributed rate variation across sites and a proportion of invariable sites, was specifically used and other parameters were kept as default.

**Figure 2:**
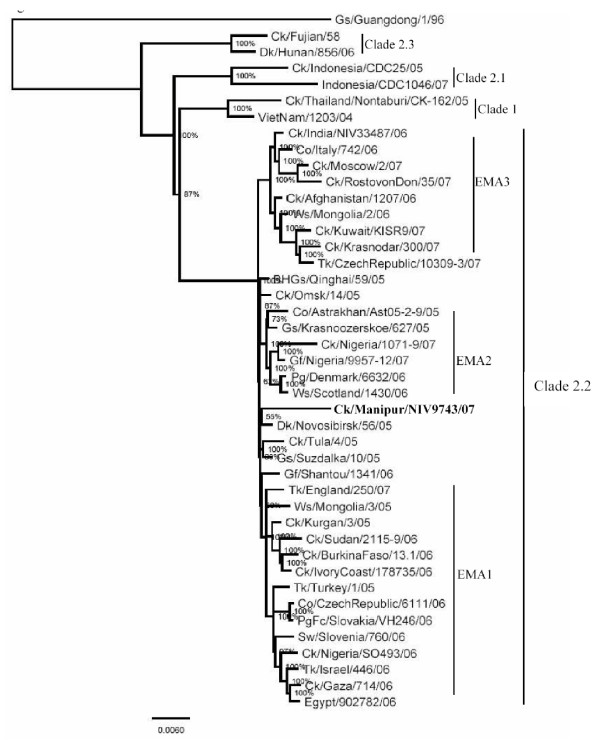
**Phylogenetic tree of whole genome (all genes concatenated as PB2, PB1, PA, HA, NP, NA, M, NS)**. Phylogenetic trees were constructed by using the Bayesian, Markov Chain Monto Carlo approach as implemented in Mr Bayes. Coding region of all genes was used for analysis. The lengths of the horizontal lines are proportional to the number of nucleotide differences per site. Scale bar indicates number of nucleotide substitutions per site. Gs/Guangdong/1/96 was used as out group sequence. Abbreviations: BHGs – Bar headed goose, Ck – Chicken, Dk – Duck, Gs – Goose, Ws – Whooper swan, Md – Mallard, Tk – Turkey, Co – Cygnus olor, Gf – Guinea fowl, Pg – Peregrine, PgFc – Peregrine falcon,

**Figure 3:**
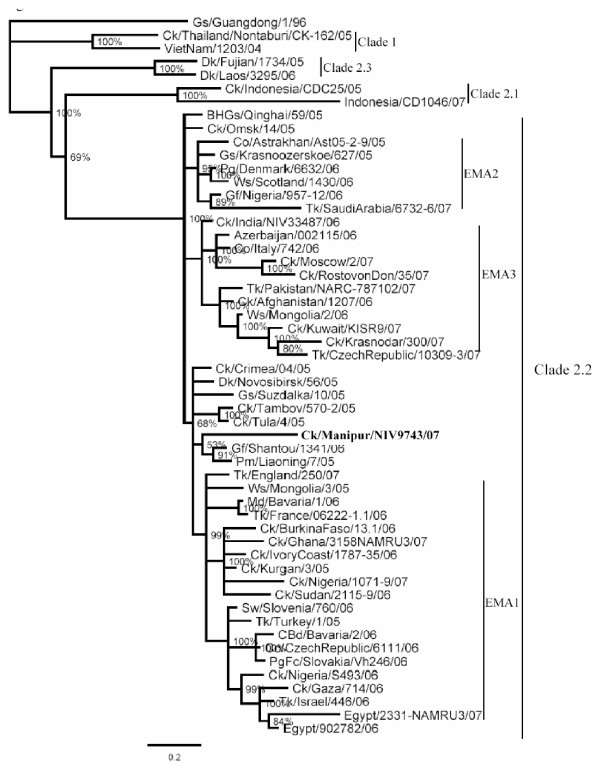
**Phylogenetic tree of HA gene**. Phylogenetic trees were constructed by using the Bayesian, Markov Chain Monto Carlo approach as implemented in Mr Bayes. Coding region of all genes was used for analysis. The lengths of the horizontal lines are proportional to the number of nucleotide differences per site. Scale bar indicates number of nucleotide substitutions per site. Gs/Guangdong/1/96 was used as out group sequence. Abbreviations: BHGs – Bar headed goose, Ck – Chicken, Dk – Duck, Gs – Goose, Ws – Whooper swan, Md – Mallard, Tk – Turkey, Co – Cygnus olor, Gf – Guinea fowl, Pg – Peregrine, PgFc – Peregrine falcon,

**Figure 4:**
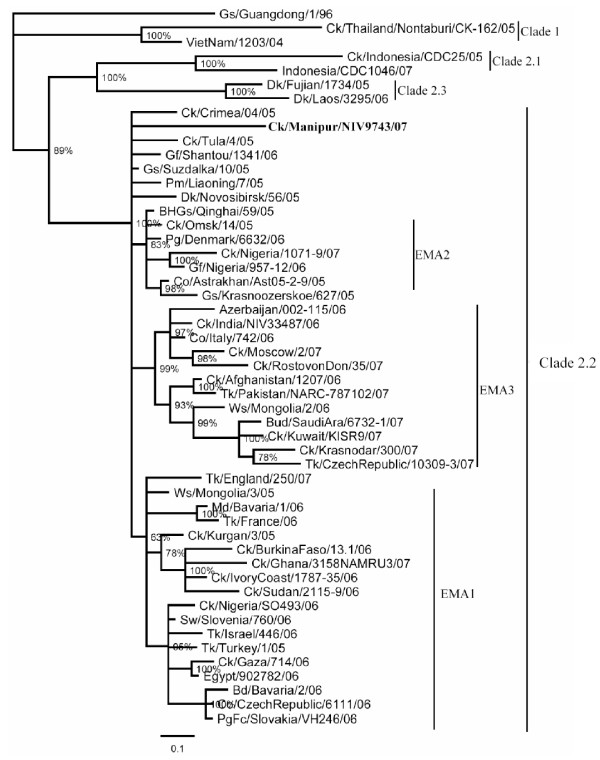
**Phylogenetic trees for NA gene**. Phylogenetic trees were constructed by using the Bayesian, Markov Chain Monto Carlo approach as implemented in Mr Bayes. Coding region of all genes was used for analysis. The lengths of the horizontal lines are proportional to the number of nucleotide differences per site. Scale bar indicates number of nucleotide substitutions per site. Gs/Guangdong/1/96 was used as out group sequence. Abbreviations: BHGs – Bar headed goose, Ck – Chicken, Dk – Duck, Gs – Goose, Ws – Whooper swan, Md – Mallard, Tk – Turkey, Co – Cygnus olor, Gf – Guinea fowl, Pg – Peregrine, PgFc – Peregrine falcon,

The GenBank accession numbers for the PB2, PB1, PA, HA, NP, M and NS gene segments of the isolate, A/Ck/India/NIV9743/07, herein referred to as the Manipur isolate, is from FJ719831–FJ719838. The percent nucleotide identity (PNI) and percent amino acid identity (PAI) values were calculated as pairwise p-distances, for a dataset of about 80 representative sequences in each gene. For clarity, limited representative sequences are shown in the phylogenetic trees (Figures 2, 3 and 4).

## Results

In Chingmeirong, Manipur, mortality was noticed in a poultry flock on 7^th ^July 2007. The birds were housed in 2 separate backyard coops, 132 in one and 12 in another, separated by just about 5 meters. All the birds of the first coop died in about 4 days. After one week, one bird in the second coop fell sick and was culled. The clinical samples were collected and sent to our laboratory for investigations. All the other birds were culled. Subsequent investigations revealed that the birds were moved from a village farm, located at the interface of forest and agricultural land, about 40 kms away from Imphal city, just a few days before mortality was noticed. It is likely that the birds might have contracted the infection in that place which is frequented by many wild birds. Samples collected from other parts of the city and in adjoining localities were all negative for the virus.

### Isolation and Identification

Virus was isolated from all six samples and in all the cases chick embryos died within 24 hours post-infection. Confluent monolayers of MDCK cell lines infected with the specimen showed high cytopathic effect. Allantoic fluid from dead eggs and tissue culture supernatants from infected cultures tested positive for avian influenza (H5N1) virus in HA and HAI.

Samples tested in one step RT-PCR showed amplification of influenza A, H5 and N1 gene specific bands. A 219 base pair band appeared showing presence of H5 and 668 base pair for the presence of N1. Real Time RT-PCR analysis showed the presence of Avian influenza (H5N1) in all the specimens (Data not shown).

### Phylogenetic analysis

Phylogenetic analysis of 41 whole genomes, all the eight gene segments concatenated, showed that the Manipur isolate was unique in the clade 2.2, as it did not cluster with the majority of the isolates in the EMA sublineages 1 to 3 (Figure 2). It was close to A/Dk/Novosibirsk/56/05 and other ungrouped isolates such as A/Gs/Suzdalka/10/05, A/Gf/Shantou/1341/06 and A/Ck/Tula/4/05.

The HA gene of the Manipur isolate grouped with A/Gf/Shantou/1341/06 and A/Pm/Liaoning/7/05 (Figure 3) outside the EMA sublineages. The highest percent nucleotide identity (PNI) of 98.73 was found to be with the A/Gs/Crimea/615/05 isolate followed by the A/Dk/Novosibirsk/56/05 isolate with PNI 98.67. The highest percent amino acid identity (PAI) was observed to be 99.27 (4 amino acid differences) with other isolates including the A/Ws/Mongolia/3/05, A/Ck/Crimea/04/05 and A/Ck/Omsk/14/05.

The NA gene of the Manipur isolate did not group with any of the EMA sublineages and maintained its position with the other unassigned viruses (Figure 4). The closest PNI was 98.81 with the A/Gs/Suzdalka/10/05 isolate, followed by A/BHGs/Qinghai/59/05, A/Ws/Mongolia/244/05, A/Tk/Turkey/1/05, A/Sw/Slovenia/760/06, with PNI varying from 98.74 to 98.66. The isolates with which it shared the minimum (nine) amino acid differences included the isolates of 2005 from Turkey, Suzdalka, Qinghai, Astrakhan, Omsk and Slovenia/06.

The phylogenetic analysis of the polymerase genes again showed that the Manipur isolate was distinct and remained unassigned with respect to the three EMA sublineages. The PB2 gene showed closest PNI (99.03) with the A/Gf/Shantou/1341/06 isolate, followed by the A/Dk/Novosibirsk/56/05 and the A/Ck/Omsk/14/05 isolates with 98.99 PNI and 9 amino acid differences in all the three cases. It showed closest amino acid identity of 98.95 (8 differences) with several isolates including A/Ck/Egypt/22531/06, A/Sw/Slovenia/760/06, A/Ck/Gaza/714/06, A/Pg/Denmark/6632/06, A/Tk/Turkey/1/05 and A/Co/Croatia/1/05. In case of the PB1 gene, the Manipur isolate was closest in PNI (98.95) to the A/Ck/Kurgan/3/05 isolate with also the highest amino acid identity (99.45%, 4 amino acid differences). The PA gene of the Manipur isolate had closest PNI (98.83) with the A/BHGs/Qinghai/59/05 isolate, followed by Azerbaijan/002115/06 with 7 amino acid differences. The closest PAI (99.16) with 6 amino acid differences was with the A/Ck/India/NIV33487/06 isolate (PNI 98.65). The Novosibirsk/05, Liaoning/05 and Mongolia/06 and Iran/06 showed PAI of 98.89 (7 aa differences) but higher PNI (98.7).

The NP gene showed closest PNI with the A/Gs/Hungary/3413/07 isolate (98.9) and 1 amino acid difference. The closest PAI (99.79) also amounting to a single amino acid difference was with several isolates including the A/Tk/England/250/07, A/Gs/Qinghai/F/06, A/Md/Bavaria/1/06, A/Turkey/15/06, A/Krasnoozerskoe/627/05, A/Omsk/14/05, A/Dk/Novosibirsk/02/05, A/Ck/Kurgan/3/05, and A/Ws/Mongolia/3/05. The M gene shared the highest PNI (98.81) with the A/Dk/Novosibirsk/56/05 isolate. The NS gene had highest PNI with A/Dk/Novosibirsk/05 and A/Suzdalka/10/05 (98.96) followed by the A/BHGs/Qinghai/F/06 isolate (98.9). Again, in all these genes the Manipur isolate was distinct from the others and also did not cluster with any of the EMA sublineages.

### Mutations

In the HA gene, among amino acid mutations, the Manipur isolate had a substitution K328R (H5 numbering and substitution mentioned with reference to A/Vietnam/1203/04, belonging to clade 1). This corresponds to the novel HA cleavage site, GERRRRKR that was originally found in three whooper swan isolates of Mongolia in 2005 (GenBank accession numbers AB233320–AB233322). Subsequently, the same pattern was noted in several isolates of 2007 from Egypt (EF535822–EF535825, EU496393) and Nigeria (EU148396). Another non synonymous mutation, S155N, at the N154 glycosylation site, observed in the Manipur isolate was also noted in the 2005 isolates of Novosibirsk, Qinghai, Tambov, Crimea, Omsk, Suzdalka, and Liaoning and majority of the EMA1 isolates. D54N in the Manipur isolate was shared with the A/Ck/Egypt/1079NAMRU3/07 isolate and the A/Ws/Mongolia/4/05 isolate. One unique mutation L297F in the Manipur isolate was not noted in any of the clade 2.2 isolates but was observed in A/Dk/HongKong/ww381/2000. T513A mutation was shared with A/Ck/Krasnador/199/06. Among synonymous nucleotide substitutions in the Manipur isolate, G42A, G705A, T861C, 983G, A1632G were shared with the Egypt/07 and/or Nigeria/07 isolates; T78C with A/Tk/SaudiArabia/67326/07; T573C with A/Gf/Shantou/1341/06, A/Pm/Liaoning/7/05 and A/Tk/CzechRep/10309-3/07; C1335T with A/Dk/Novosibirsk/56/05 and A/Ck/Sudan/21159/06.

In the NA gene, the Manipur isolate had 1 unique amino acid substitution A285T that is not observed in any of the H5N1 NA sequences. V264I, a unique mutation with respect to clade 2.2 isolates was found in several Guangxi/06, Indonesia/06 and Vietnam/04 isolates. Among synonymous nucleotide substitutions, 1018T was as in A/Tk/Turkey/1/05 and A/Ck/Nigeria/10719/07 along with Vietnam/1203/04, while T1332C was as in A/Ck/BurkinoFaso/13.1/06.

The Manipur isolate showed two unique amino acid mutations, K116R and I411M, in the PB2 gene, that were not observed in any of the available H5N1 sequences. V366I was shared with A/Dk/Egypt/22533/06. Notably there were five unique amino acid mutations E60V, A142S, K197R, K737E and M744I in the PB1 gene of the Manipur isolate. M317I mutation was shared with Indonesia/07 and Vietnam/05 isolates, while K745R mutation was shared with A/Gf/Shantou/1341/06 and A/Ck/Liaoning/7/05. Among synonymous nucleotide substitutions, G504A was shared with Qinghai/05, Krasnadar/07 and CzechRep/07 isolates; and G1437A with Kuwait/07 isolates. In the PA gene, mutations P332L was as in A/Ck/Scotland/1959; E351G in A/Partridge/Shantou/1075/02; D396E in A/Dk/Hunan/1204/06 and T618A in A/Grebe/Novosibirsk/29/05 and Azerbaijan/06 isolates. Two unique mutations, R213I and A598S were also observed in the PA gene of the Manipur isolate.

A synonymous substitution, A91C, in the NP gene, was unique and not observed in any of the available H5N1 sequences. In the M gene, V68I of the Manipur isolate was shared with A/Ck/Nigeria/104754/06 and RostovonDon/07 isolates. The non synonymous mutations included, A408T shared with A/Dk/Novosibirsk/56/05; G480A with A/Ck/Nigeria/10719/07; G890A with A/Ck/Nigeria/104754/06 and Rostovon/07 isolates. In the NS1 gene, two unique mutations, V111M and L212P, were observed while a unique mutation, F55L, was observed in the NS2 gene.

The gene coding for the PB1-F2 protein in the Manipur isolate, was observed to have two nucleotide polymorphisms, A85T and G86A. These polymorphisms resulted in the introduction of a stop codon at position 29 in the PB1-F2 that was unique to the Manipur isolate. Similar to this, a stop codon was noted in A/Gf/Shantou/1342/06 at position 25. Another nucleotide substitution, C123T in PB1-F2 of the Manipur isolate was observed only in clade 2.1 isolates. An amino acid substitution, G110A was shared with A/Co/Croatia/1/05 and A/Ck/Nigeria/10719/07.

The amino acid variations observed in the Indian isolates, for all the eight gene segments and the PB1-F2 protein are indicated in Table 1.

**Table 1:** Amino acid variations between Ck/India/NIV33487/06 (India 06) and Ck/India/NIV9743/07 (India 07).

Protein	India '06	India '07	Protein	India '06	India '07
**HA**			**PB2**		
54	D	N	116	K	R*
155	D	N	126	R	K
297	L	F	195	D	N
328	K	R	338	I	V
513	T	A	366	V	I
			390	N	D
**NA**			411	I	M*
34	V	I	451	T	I
44	R	C	483	M	T
72	T	A	494	V	I
264	V	I			
267	I	V	**PB1**		
285	A	T*	60	E	V*
287	E	K	142	A	S*
334	S	N	197	K	R*
340	P	S	317	M	I
398	D	E	737	K	E*
			744	M	I*
**NP**			745	K	R
10	H	Y			
52	Y	H	**PA**		
397	S	N	213	R	I*
			332	P	L
**M2**			351	E	G
68	V	I	396	D	E
			598	A	S*
**NS1**			618	T	A
111	V	M*			
207	G	D	**PB1-F2**		
212	L	P*	37	R	Q
			48	P	Q
					
**NS2**					
50	V	M			
55	F	L*			
60	T	I			

Unique mutations in Manipur 2007 indicated with an asterisk

### Molecular markers

Of the 14 residues reported to form the receptor-binding pocket in HA1 [19], two substitutions K193R and R216K were observed in the Manipur isolate. The residues that preferentially binds the avian-like α2,3-NeuAcGal linkages, G225, S227, G228, Q226, S221 and R216 were conserved. Host specific amino acid residues [20] in the PB2, PA, NP, M1 and M2 proteins, all suggested avian specificity except V28 in the M2 protein, known for adaptation to human hosts. The isolate possessed Lys at position 627 of the PB2 gene, which is associated with increased virulence in mammals [21]. Of the known markers for enhanced polymerase activity [22], only one substitution, L13P in PB1 protein is observed in the Manipur isolate. The PB1-F2 protein, that has been recognized as a virulence contributor and functions in reducing the immune responses as well as increasing the cytotoxicity [23,24], was observed to possess a stop codon at position 29. Similar to this, a stop codon was noted in Gf/Shantou/1342/06 at position 25. The C-terminal protein was conserved when compared to other PB1-F2 proteins except for a P48Q mutation (Table 1). E92 in the NS1 protein, implicated in human adaptation, was observed in the Manipur isolate. The C terminal four amino acid stretch corresponding to the PDZ-domain ligand motif in the NS1 protein was ESKV and is reported as being specific to avian isolates [25].

The NA sequence did not show any mutation conferring resistance to oseltamivir [26]. Similarly, no mutation was observed in the M2 ion channel that conferred resistance to amantadine [27].

## Discussion

The EMA 1–3 sublineages represent the influenza (H5N1) viruses isolated in Europe, the Middle-Eastern region and Africa beyond 2005 [12]. A minority of isolates, such as those of Qinghai, Novosibirsk region, Shantou and Omsk did not group with either of these sublineages and had been left unassigned. On the basis of the Bayesian phylogenetic analysis in this study, in all the 8 gene segments, the Manipur isolate grouped with the unassigned viruses in the EMA nomenclature.

The molecular characterization of the viral proteins of the Manipur isolate was carried out for pathogenicity markers and sensitivity to antivirals. The HA of the Manipur isolate had the novel cleavage site (GERRRRKR) that was first reported in three whooper swan isolates in Mongolia, 2005 and also in the recent Nigeria/07 and Egypt/07 isolates. Though variations in the cleavage site such as RERRRKKR, RERRKKR and RERRRR have been linked to human H5N1 cases [28], no such report exists for GERRRRKR motif. Hence the significance of this cleavage site remains to be understood. D54N mutation in the Manipur isolate, results in an additional putative glycosylation site, the implication of which is not yet known. The two substitutions in the HA1 receptor binding domain were also noted in other clade 2.2 viruses including our 2006 isolates and may not have implications in modifying avian associated α2–3 linked sialic acid specificities. Similarly, no marked mutations have been observed in either of the PB2, PA, NP, M1 and M2 proteins with respect to enhanced polymerase activity or host specificity. Sensitivity to antivirals was also noted in the Manipur isolate. Notably there was a stop codon in the PB1-F2 protein of the Manipur isolate. On one hand, protein knock-out studies have shown that the C-terminal region of PB1-F2 can be expressed from a downstream initiation site [24], implying that the protein of the Manipur isolate may still get expressed from a downstream initiation codon. Further, though the C-terminal region of the PB1-F2 protein, known to be responsible for major functional roles such as mitochondrial localization and PB1-F2 induced apoptosis [29,30] was conserved, the variations observed in the Manipur isolate may be of interest and their significance is yet to be ascertained. Truncation of the PB1-F2 protein at position 29 on the other hand could have critical consequences. Recent reports [31] have shown that non expression of the PB1-F2 during infection results in an altered localization of PB1 and decreased viral polymerase activity. In H1N1 viruses, truncated PB1-F2s reported due to in-frame stop after codons 11, 25 and 34 have been correlated with an inefficient polymerase complex and lesser epidemic severity [32,33]. Thus, the stop codon in Manipur PB1-F2 protein might have implications on the replication efficiency and may have a possible role to play in the focal nature of the outbreak. A functional PDZ-binding domain is suggested to correlate with human virulence and human H5N1 isolates generally have a motif, RSKV in the NS1 protein [25]. The effect of the ESKV motif as in the Manipur isolate and other avian isolates, is still unknown, though it has been shown to be a potent type I PDZ-binding domain in other systems [34,35].

The phylogenetic characterization of the Manipur isolate demonstrated that the virus isolated in 2007 in India was distinctly different from the viruses of the three EMA sublineages. Considering that all the eight genes of the 2006 isolates of India belonged to the EMA3 sublineage, with several mutations observed between the two strains, the possibility of local evolution can be excluded. Genetic analysis of the Manipur 2007 isolate clearly indicates a second introduction of the H5N1 virus in India. Among the other isolates that have been placed outside the EMA groups, the Manipur isolate was distinct and unique. It was related to viruses isolated from wild migratory waterfowl from Russia (Novosibirsk, Suzdalka), China (Shantou, Liaoning, Qinghai) and Mongolia. A recent report [36] stated that the H5N1 isolate from the Bangladesh outbreak belonged to subclade 2.2 of the Qinghai lineage and was most closely related to viruses isolated from Afghanistan, Mongolia and Russia [37]. Further, as seen from Figs 2, 3 and 4 these isolates fall into EMA3 and are closely related to the 2006 isolate from India. Thus the Manipur virus is not related to the virus prevalent in Bangladesh. The uniqueness of the Manipur isolate was brought out in terms of its own specific amino acid and nucleotide polymorphisms. The Manipur isolate had 13 unique amino acid substitutions in the eight genes. The acquisition of polymorphisms as seen in other recent isolates of 2007 from distinct geographical locations such as Nigeria, Egypt, Saudi Arabia, Kuwait etc along with those from earlier outbreaks in 2005–06 in Turkey, Crimea, Novosibirsk, Mongolia and Qinghai suggests the possibility of transportation of H5N1 viruses through migratory birds. In terms of the recent acquisitions as in Nigeria and Egypt, interestingly, the timeline [38] shows that the said Nigerian outbreaks were first reported in September 2007, after the Manipur outbreak in July 2007. This eliminates the direct involvement of the former strains and points to an origin of these mutations probably in the Russian federation where the congregation of birds is known to occur [39]. Recent reports [40] on the H5N1 viruses in poultry in the Russian Federation, have proposed that the viruses may be genetic reassortents of the Qinghai-like viruses likely to have been introduced into Russia from China by migrating birds. Further, the inclusion of mutations from 2005–06 isolates of Turkey, Mongolia, China, Russia/Siberia (Novosibirsk, Suzdalka, Crimea) indicate the role of the migratory birds spreading from the Russian federation into the Central Asian and Black sea/Mediterranean flyway. Cross over of migratory birds in the flyway intersection has been occurring [41] and thus new variants might have got into central Asia. Though the exact, source of the introduction into India cannot be concluded, considering that similar strains have not been reported during the period of the Manipur outbreak in the neighbouring countries of Pakistan, Afghanistan, Bangladesh, and Myanmar, it appears that there may have been a independent introduction into the country. Existing reports, that, migratory waterfowl from the Qinghai lake migrate southwards to Myanmar and over the Himalayas to India, around September returning to the Qinghai lake around April [41], support such an assumption.

## Conclusion

Overall, our findings suggest that the Manipur 2007 virus isolate is a unique variant and not related to the 2006 Indian isolate. The introduction of such a virus, either directly or indirectly, into India calls for improved surveillance in the country and subcontinent. The appearance of such variants is also serious concern for the emergence of even more highly pathogenic strains and a pandemic threat.

## Abbreviations

BHGs: Bar headed goose; Ck: Chicken; Co: Cygnus olor; Dk: Duck; Gf: Guinea fowl; Gs: Goose; Md: Mallard; Pg: Peregrine; PgFc: Peregrine falcon; Pm: Piedmagpie; Sw: swan; Tk: Turkey; Ws: Whooper swan.

## Competing interests

The authors declare that they have no competing interests.

## Authors' contributions

ACM, AKC and SDP conceived and designed the experiments. AKC, SDP, BP, SR, SK and SSK performed the experiments, SSC, SMJ and AKC planned and performed the bioinformatics studies, SSC, AKC, SMJ and ACM analyzed the data, SSC, AKC and ACM wrote the paper. All authors read and approved the final manuscript.
